# Editorial and Miscellaneous

**Published:** 1865-01

**Authors:** 


C. S. Medical & Surgical Journal.

RICHMOND, JANUARY, 1865.

*E. W. AYRES............PUBLISHER AND PROPRIETOR.*

### EDITORIAL AND MISCELLANEOUS.

A year of storm and tempest, of war and alarms has swiftly passed away, and, in offering the usual compliments to our readers, we may be pardoned a few reflections not foreign to the occasion.

We may be allowed to congratulate ourselves that the Journal, founded under so many adverse circumstances and surrounded with so much to impede its early progress, has survived the year, which has just departed. Under the pressure and exigencies of the times, the scarcity of material and the want of labor, every literary and scientific periodical of the South has been suspended, and most of them have ceased to exist. Our little bark, alone, has safely weathered the gale, and commences its voyage anew, in good heart for the future.

The circulation of the Journal has surpassed any reasonable expectation, for such times as these surely do not favor the growth and cultivation of scientific studies, and we therefore may note with some pride that our readers are more numerous than ever honored a similar publication in our country, and, we have reason to think, that but for the want of proper post-office facilities, the subscription list would receive many accessions.

It is also a subject of remark, that in spite of the rigorous blockade, the unceasing efforts of our enemies to deprive us of all intercourse with foreign countries, the Journal has never failed to have access to the most recent foreign periodicals which, while they have lacked in variety, at least enable us to offer to the reader an epitome of all the valuable material being collected in more peaceful countries, where nothing disturbs the daily progress in scientific research and observation.

And to the critics, who may undervalue our modest labors, and who, indeed, have but little trouble in pointing out many shortcomings; who smile at the sometimes wild propositions of our enthusiastic correspondents, or sneer at the dry statistics of hospital records—to these we would say, and with truth, that we confidently look to them in the coming year to relieve the Journal from such well founded censures. With so much food for study, such opportunities for observation, so great a field for practice, it would be indeed a reproach to our profession if it failed to make the original department of their only medical periodical rich with useful matter. The invitation to co-operate with the editor of this Journal is again earnestly extended to the physicians of the South, who alone can place our work on the elevated ground that its friends desire so much to see it assume. If the Journal fails, its conductors have determined that the fault shall be laid to the door of the Southern profession, who refuse to bestir themselves in spite of every inducement; to shew themselves true lovers of their calling, both able and willing to overcome all obstacles, and, in the midst of tumult, with quiet minds to ponder over the vast fields of knowledge which surround them. Whatever fate betide us, let us be faithful to the last to our noble art, and come what may, the satisfaction of duties faithfully performed, obligations fairly met, and responsibilities honestly borne, will be ours.

### *Foreign Honors to an Army Surgeon.*

The recent inauguration of the statue to Baron Larrey in the town of Tarbes is suggestive of considerations which, in the present condition of our Army Medical Department, may be deserving of expression. The ceremony was accompanied by all the display peculiar to France. The place of Larrey's nativity now boasts of the distinction, and presents to posterity a monument to the memory of one, great amongst great men, who, in the service of science and the pursuit of his noble profession, by the independent exercise of those rare abilities with which Providence had endowed him, raised himself to a position as it was deserved. To trace the history of Larrey, and to follow him step by step through the dangers he braved throughout his distinguished career, would be to detail the wars of the First empire. It is enough to say that by his singular devotedness to surgical science, by the combination of rare gifts of genius and discretion, of daring and reserve, Larrey became the idol of the French army and the companion and friend of the Emperor, whose last expression respecting him constitutes his proudest epitaph. This is not the only monument to his memory. A statue in bronze, by David d'Angers, the famous sculptor of those of Ambrose Pare and Bichat, cast from cannon taken in the different great battles in which the renowned surgeon distinguished himself, was risen to his memory in Paris by subscription; and Marshal Soult, Duke of Dalmatia, then Minister of War, decided upon its being erected in the *cour d'honneur*, the grand square of the Hopital du Val de Grace. Larrey is represented in his uniform, pressing to his heart the will of Napoleon, on which is inscribed the words "C'est le plus vertueux et le plus honnete homme que j'aie connue." These are but the perishable evidences of his greatness, and the least durable of its records. The student of military surgery must set before him as an example worthy of imitation, Baron Larrey, Surgeon-in-Chief of the great French army, and Baron of the Empire—one who created for himself a monument which must last for all time. To his genius are due many of those practices now received and adopted as indicating the true principles which should guide the military surgeon in active service. The "ambulances volantes," first introduced by him when acting as surgeon-major to the hospitals of the army of the Rhine, have since done good service on the battle-field, and are now universally employed. His writings on military surgery must ever be regarded as text-books on the particular subjects of which they treat. His bold vindication of the independence of his profession, and his manly advocacy of the immunities which sickness and the casualties of war entail, are matters of history. These constitute his paramount claims to distinction. It is difficult to determine where he was most distinguished: whether at Aboukir, where for his coolness and courage in operating upon many under fire he was presented by Napoleon with a sword having the words "Larrey" and "Aboukir" engraved upon it; or fifteen years later, when at Bautzen he vindicated the wounded from the grave imputation of self-mutilation and braved the Emperor's wrath, not without convincing his judgment, again to receive a noble recompense for the independent discharge of the high duties with which he was entrusted. We might follow Larrey through the campaigns of Germany, Prussia, Poland, and Spain, where he filled the office of Surgeon-in-Chief to the Imperial Guard, until, on the field of Wagram, he was raised to the distinction of Baron of France. Few of our readers are unacquainted with these details, we therefore forbear to enter upon them.

"What a splendid career!" one exclaims: "and what a noble country which admitted of his being so honored! No English surgeon could have achieved a similar position." True: but remember the occasions and the man. How many Larreys could our military service boast? Admit that in personal courage we could find his equal; that for zealous devotion to the duties of their profession numerous examples are to be met with amongst those whom the Horse-Guards no longer care to honor; and that in the practical knowledge of their profession, as well as in the manual dexterity essential for its successful exercise, our army surgeons could supply many equally entitled to confidence. It is in the combination of such qualities and their active employment that Larrey founded his permanent reputation. Have our army surgeons of the present day taken such steps to "do likewise" as might reasonably have been expected of them? Grant that the position of the English army surgeon is one which now especially attracts much attention. Admit that the authorities, by a series of contemptible quibblings, have disgusted many with a position in itself highly honorable, but rendered intolerable through their narrow-minded vacillation as to the status of the medical officer, and their apparent inability to determine if he should occupy the position of a gentleman equally with those whose business it is to kill rather than to cure. Admit all this, and deplore it. We yet ask, does the English military surgeon, as a rule, fulfil the highest expectations which his responsibilities and opportunities warrant? How many record their experience? How many suggestions on military hygiene have the profession or the service been favored with from those whose life ought to be devoted to such pursuits? What results have followed from the extraordinary experience of modern warfare? Are our encampments regulated by sounder views of that which is essential for health? Is the treatment of the wounded provided for with more complete regard to the necessities of their position? Are the ravages of epidemics anticipated and prevented through more effective arrangements and discipline? Is the control of the Army Medical Department distinguished for those many improvements in detail which the light of modern science has revealed? Or does recent experience prove that there is yet room for progress? These are questions we commend to the careful reflection of those to whom great opportunities present themselves. High positions involve commensurate responsibilities; and what position is more important than that of the military surgeon? We have often deplored the silence on matters professional which characterizes the Army Medical Department, including as it does many of the most exalted genius and largest experience. Larrey found time to place on record the achievements of the one, guided by the observation of the other. We commend his example to all, and bid them do likewise.

---

*To Ascertain the Purity of Chloroform.*—If a small piece of sodium is thrown into pure chloroform, no action whatever ensues; if, on the contrary, any impurity, such as alcohol, be present, then a disengagement of gas takes place.

*London Punch on Army Surgeons.* From an Army Swell.

Pray don't imagine, *Punch,* that the Surgeon-Famine in the army is the fault of the swells. I suppose I am what is called a swell. My ancestors came in with CANUTE. They have never exercised any branch of industry, and have always lived sumptuously on the labor of others. I myself am in the army, simply because I think I ought to be something more than a swell, and am fit for nothing else so much as for a soldier.

Now, of all the fellows in a regiment, I assure you, I consider the surgeon to be, generally, the most of a gentleman. He is at least as much of one as any of them, and he has, if regularly appointed, been made as much more of a gentleman than the rest as a much better education than they have had could make him. The indignity which army surgeons are treated with proceeds not from pride of rank and birth on the part of any of their brother officers, but from a consciousness of the want of those advantages on the part of some of them. In this commercial country many a fellow enters the army who never had a grandfather that he could give any account of, and the best such a fellow can say of his pedigree usually is that his father was a tailor. More commonly an officer of that class of fellows is the son of a large mercantile rogue, or a swindling railway jobber. Well, he cannot help that; and he is rich, and his own money at least was not ill-gotten; and he might be a gentleman if he chose. But instead of that, he is too often a purse-proud snob. This is the sort of fellow that thinks it necessary to assert his position by insisting on the abasement of army surgeons. It is not the swells in a regiment, *Punch,* who are insolent to the surgeon, but only the snobs. Mushrooms these snobs are called by men who have less respect for a mushroom than I have, for I consider it an excellent ingredient, not an objectionable one, in a mess. Those who term them mushrooms, will further say that, inasmuch as they peculiarly abound in the cavalry, the majority of them are horse mushrooms; but, comparing these bloated and extremely offensive snobs to fungi, I would rather name them toadstools.

I consider the surgeon quite as much a combatant officer as myself. We don't in these days, charge with lances in rest, and we no longer brandish battle-axes and maces. He is as likely to be struck down at any time by disease, sometimes by shot, as I am. I wish no invidious distinction to be made between him and myself. I would not assign him the uniform of a beadle. Let him wear that of his relative rank in the army, or be allowed to dress in plain clothes, so that he might, as perhaps he would like to, be distinguished from a combatant fool.

Unless the reasonable demands of the army surgeons are granted, I shall be obliged to throw up my commission. Suppose I am killed in action, well and good. I am prepared for that. But I may be wounded. For that I am prepared too. I am always ready to lose a limb for my country. But my country must take care that it shall be skilfully amputated. I expect my country to provide that any operation which its service may require me to undergo shall be performed safely, quickly, and pleasantly, as much as it can be. Certainly I value my blood too highly to allow it to be spilt by a bungling operator. I don't at all relish the idea of an acting-assistant surgeon, obtained by advertisement, attempting to extract a bullet deeply lodged in the complicated anatomy of your humble servant,

ARMIGER.

*Rag and Famish, Sept.* 1864.

---

*Hippophagy Again.*—One of the secretaries of the French Society for the Protection of Animals, in a lecture given the other day in Paris at the Garden of Acclimatation, revived the proposal to constitute horseflesh an article of food, demonstrating its acceptability with a tureen of horse soup, and another dish of that noble animal dressed *a la daube*, which he offered to his audience, and they, including many ladies, devoured. Well; who shall tax them with eating strange food? If all the prime tongues ready cooked, on sale at our British grocers could speak, and would tell the truth, we apprehend that not a few of them would neigh.

There is support, doubtless, in saddle of horse, but, for eating, we are disposed to prefer saddle of mutton.—*Punch.*

